# Editorial: Genetics of domestication and diversification towards evolution of crop plants

**DOI:** 10.3389/fgene.2023.1175931

**Published:** 2023-03-23

**Authors:** Aditya Pratap, Prakit Somta, Petr Smýkal, Sanjeev Gupta

**Affiliations:** ^1^ ICAR-Indian Institute of Pulses Research, (ICAR), Kanpur, India; ^2^ Department of Agronomy, Faculty of Agriculture at Kamphaeng Saen, Kasetsart University, Nakhon Pathom, Thailand; ^3^ Department of Botany, Faculty of Science, Palacky University, Olomouc, Czechia; ^4^ Indian Council of Agriculture Research, New Delhi, India

**Keywords:** domestication, crop evolution, evolution, neo-domestication, QTL

Domestication has been a unique and the most intense form of biological evolution leading to the establishment and propagation of newly domesticated species. In the past 12,000 years, it has brought novel plant and animal species into human cultivation, adding new food, forage, ornamental, and companion species to our usage. The process of crop domestication is primarily based on selection driven by human cultivation practices and agricultural environments (von Wettberg et al., 2020). Approximately 2,500 species have undergone some degree of domestication, and 250 species are considered to be fully domesticated (Meyer et al., 2012). Domestication of crop plants has been largely influenced by their economic and cultural importance for humans and their progression and subsequent evolution are dictated by the genetic architecture of the plant, its ecology, nature of propagation, and breeding behavior. Among all living organisms, crop plants in particular have benefitted the most from the evolutionary progression primarily associated with domestication. Domestication brings extensive evolutionary changes in the genetic architecture that augment the fitness of a new plant species under human management, simultaneously decreasing their fitness in the wild, sometimes making it completely dependent on humans for survival. It spans a wide range of events during the evolution of crop species, including initiation of evolutionary divergence from wild ancestral species and subsequent changes in appearance and ecology. Nonetheless, crop wild relatives (CWR) fortunately still exist in nature and are becoming more important in contributing to the further evolution of the domesticated species by donating their useful chromosome segments through natural and planned introgression, also offering opportunities for neo-domestication (Pratap et al., 2021). The need to highlight the importance of domestication and the underlying processes provided the impetus for this Research Topic.

Understanding the evolutionary adaptation of a crop species requires an understanding of the ecology of their wild ancestors and the selective pressures that farmers exerted when they started domestication and shaping agricultural environments. The Research Topic of genetic and environmental interactions has been addressed by Cortés et al. who deployed a novel, interdisciplinary approach to combine ecological climate data with evolutionary genomics. Genome–environment associations (GEA) behave like traditional genome-wide association studies (GWAS), but instead of modeling a set of phenotypic traits, these associations consider environmentally derived variables. The current limitation is the availability of genomic characterizations of CWR, landraces, and orphan crops that span contrasting habitats. This would offer a straightforward scenario to identify natural standing adaptation to abiotic pressures such as drought and heat stresses.

Food legumes are a key component of agricultural ecosystems. There are numerous well-documented benefits of legume cultivation, especially their significant role in nitrogen fixation and improvement of soil fertility, in addition to meeting the demands of the human population for protein and energy. The review by Ambika et al. provides a view of the origin, domestication history, and diversification of major legume crops. It lists the currently known and identified loci and candidates as well as functionally proven genes governing domestication traits. This is followed by examples of various strategies to either harness existing diversity conserved in gene banks or generate new variations through mutagenesis or gene editing.

Through genome-wide association studies (GWAS), candidate gene studies, quantitative trait locus (QTL) mapping and cloning, whole-genome sequencing, and transcriptome analysis, domesticated species have been the subject of molecular studies over the past couple of decades in order to identify genes associated with the main agronomic traits, as well as domestication-related traits. Yuan et al. reported the results of transcriptomic profiling of cultivated (*L. culinaris*) and wild lentil (*L. orientalis*) genotypes during the shade response. Although the gene expression profiles of wild and cultivated genotypes were similar, namely, the upregulation of genes involved in gibberellin, brassinosteroid, and auxin synthesis and signaling pathways, as well as cell wall modification related to the stem elongation response under the shade, there were differences too. Wild lentils showed downregulation of the genes involved in jasmonic acid and flavonoid biosynthesis pathways, and upregulation of defense response genes.

The genus *Vigna* is an important taxon of leguminous plants comprising ten domesticated species, some of them being semi-domesticated and others fully domesticated. *Vigna* crops are major sources of protein and carbohydrates for those living in the tropic and subtropical regions of Asia and Africa (Pratap et al., 2014). The review of Verma et al. comprehensively discusses the origin, distribution, production, and genetics of domestication of *Vigna* crops. Although these crops are domesticated in different regions, the QTL locations of domestication-related traits appeared to be clustered and conserved among different species. Domestication followed by modern plant breeding practices generally reduces the diversity of crop plants and narrow genetic diversity can result in susceptibility to insects and diseases. Bruchids or seed weevils are the most devastating insect pests of legumes after harvest. Chen et al. constructed a high-density SNP-based linkage map of mungbean and utilized it for locating the *Br* locus conferring a complete resistance to azuki bean weevil (*Callosobruchus chinensis*), the most important bruchid species of Asia, in wild mungbean (*V. radiata* var. *sublobata*) accession TC 1966. They demonstrated that the *Br* locus was on chromosome 5 and *Vradi05g03810* encoding a resistant-specific protein is the candidate gene for the resistance.

The tetraploid upland cotton (*Gossypium hirsutum*) accounts for approximately 95% of worldwide cotton production. Niu et al. followed the genetic mapping of lint percentage as one of the most important determinants of cotton yield. This is a quantitative trait with high heritability, and the authors identified 417 QTLs localized on 26 chromosomes. More than 60 QTLs were stable, and major effective QTLs can be deployed in marker-assisted selection (MAS). Using available cotton genomic resources, this research led to the identification of 90 candidate genes, providing a basis for further studies.

Overall, the articles published in this Research Topic provide insight into the domestication and diversification of crop plants, highlighting the domestication history of some of them, the development of new markers, and useful marker-trait associations. The information included in these articles could be further deployed toward the genetic improvement of crop plants through an introgression breeding approach.
